# The role of littoral cliffs in the niche delimitation on a microendemic plant facing climate change

**DOI:** 10.1371/journal.pone.0258976

**Published:** 2021-10-22

**Authors:** Miguel R. Ferreira, Alice Maria Almeida, Celestino Quintela-Sabarís, Natália Roque, Paulo Fernandez, Maria Margarida Ribeiro

**Affiliations:** 1 Departamento de Recursos Naturais e Desenvolvimento Sustentável, Escola Superior Agrária, Instituto Politécnico de Castelo Branco, Castelo Branco, Portugal; 2 C4—Centro de Competências em Cloud Computing (C4-UBI), Universidade da Beira Interior, Covilhã, Portugal; 3 Departamento de Edafoloxía e Quimica Agrícola, Facultade de Bioloxía, Universidade de Santiago de Compostela, Santiago de Compostela, Espanha; 4 QRural—Qualidade de Vida no Mundo Rural, Unidade de Investigação e Desenvolvimento do Instituto Politécnico de Castelo Branco, Castelo Branco, Portugal; 5 MED—Mediterranean Institute for Agriculture, Environment and Development, Universidade de Évora, Pólo da Mitra, Évora, Portugal; 6 CEF—Centro de Estudos Florestais, Instituto Superior de Agronomia, Universidade de Lisboa, Lisboa, Portugal; 7 CERNAS—Pólo de Castelo Branco do Centro de Estudos de Recursos Naturais, Ambiente e Sociedade, Escola Superior Agrária, Instituto Politécnico de Castelo Branco, Castelo Branco, Portugal; University of Molise, Isernia, ITALY

## Abstract

Obligate coastline taxa generally occupy very limited areas, especially when there is a close affinity with a specific coast type. Climate change can be a meaningful threat for them, reducing suitable habitat or forcing migration events. *Cistus ladanifer* subsp. *sulcatus* is an endemic plant of Portugal, known to occur only in the top of its south-western coast’s prominent cliffs. In spite of being included in the annexes II and IV of the European Habitats Directive of Natura 2000 Network, this taxon is still understudied, especially regarding the effects of climate change on its distribution. To overcome such gap, Maxent was used to model the current distribution of *C*. *ladanifer* subsp. *sulcatus* and project its future distribution considering different General Circulation Models, periods (2050 and 2070) and Representation Concentration Pathways (4.5 and 8.5). The results suggested an extensive range contraction in the future, and extinction is a possible scenario. The proximity to littoral cliffs is crucial for this plant’s occurrence, but these formations are irregularly distributed along the coast, hindering range expansions, further inhibited by a small dispersal capacity. *C*istus *ladanifer* subsp. *sulcatus* will probably remain confined to south-western Portugal in the future, where it will continue to face relevant threats like human activity, reinforcing the need for its conservation.

## Introduction

The Mediterranean Basin has long been considered crucial for plant conservation [1] due to its high levels of species richness and endemism, unsurprisingly giving it the label of biodiversity hotspot [2]. This region embodied several refugia during the climatic changes of the late Quaternary [3–5], where different taxonomic groups were concentrated and had the chance to accumulate an immense genetic diversity over time [6]. As climatic conditions grew milder, the Mediterranean landscape started dominating the Iberian Peninsula, one of the most biodiverse areas across the hotspot [1], developing a wide range of habitats and heterogeneous communities [7].

Scrublands have an active role in the Iberian ecosystems, in particular over post-fire landscapes, with *Cistus* species being frequently dominant. The western part of the Mediterranean Basin harbours most of these species [8], including the rockrose *Cistus ladanifer* L., native from Morocco, Algeria, South of France and Iberia [9]. This species exhibits high intraspecific diversity with considerable population differentiation [10] and three recognized subspecies (*africanus*, *ladanifer* and *sulcatus*) [8, 11], whose distributions converge in the Iberian Peninsula’s extreme south, denoting the importance of this refuge area (alongside northern Africa) for their evolutionary history [5, 9, 10].

*Cistus ladanifer* subsp. *sulcatus* (Demoly) P. Monts is endemic of the south-western coast of Portugal, the so-called Vicentine coast, a region characterized by mild temperatures and northerly winds carrying sea salt towards the land. This oceanic influence contributes to the diverse phytosociological associations unique to this area, many of which including *C*. *ladanifer* subsp. *sulcatus*, particularly *Genisto triacanthi-Cistetum palhinhae*, in which it is dominant [12]. Among the endemic taxa occurring in the Vicentine coast, this plant is the one with the most representative distribution along cliff tops, and thus can be considered a good proxy for the flora of this region. Lacking long-distance dispersal mechanisms [9], this plant will probably migrate towards new areas with very limited success. Hence, if its ecological niche becomes jeopardised in its current narrow range due to climate change and anthropogenic land use (e.g. increase of urban areas, croplands or tourism [13]), the extinction risk may be intensified in the future.

With the emergence of a wide range of modelling techniques and the adequate spatial data currently available, ecological niche models (ENMs) have been increasingly employed to understand taxa’s current distributions and predict range changes over future conditions [14]. ENMs are particularly important for microendemic taxa, though potentially affected by the little data generally available for them [15]. This aspect can be exceeded by designing robust modelling frameworks following the most recent advances in this field [14] and including spatially explicit data arising from novel technologies known to improve ENMs [16, 17] and ultimately biodiversity conservation [18, 19]. Remotely sensed digital terrain models with high resolution are one example that may be relevant for specialist taxa such as *C*. *ladanifer* subsp. *sulcatus*. The inclusion of cliff tops as an environmental variable in the ENM has the potential to substantially improve it, resulting in better predictions for this plant’s distribution.

The preservation of unique lineages circumscribed to highly restricted areas, as is the case of *C*. *ladanifer* subsp. *sulcatus* [20], is an unquestionable priority. Carefully designed ENMs can provide conservation stakeholders the information they need for adapting strategies focused on this plant (and more generally on the flora of the Vicentine coast) to the long term. Along these lines, the objectives of the present study were to (i) predict the current distribution of *C*. *ladanifer* subsp. *sulcatus*, identifying the most influential environmental variables; (ii) project its distribution for different future periods with several General Circulation Models (GCMs) and assess range changes over time; and (iii) analyse the implications that may arise from dispersal constraints inherent to this narrowly endemic plant’s biology, discussing consequent conservation challenges.

## Material and methods

### Taxon

*Cistus ladanifer* subsp. *sulcatus* is a woody shrub (up to 200 cm), morphologically similar to its sister subspecies *Cistus ladanifer* subsp. *ladanifer* L., although with more discernible leaf form and venation [11] and a distinct ecophysiological strategy [21]. *Cistus ladanifer* subsp. *sulcatus* occurs in coastal limestone soils and faces extreme environmental conditions [13].

This plant was recently assessed as “Least Concern” for its extinction risk by the Portuguese Red List of Vascular Flora, a category that was justified by the absence of threats that could result in significant population declines shortly [22]. It is also listed in the annexes II and IV of the European Habitats Directive of Natura 2000 Network. Even so, there is an ongoing degradation of its habitat due to human activities [13].

### Study area

**Fig 1:**
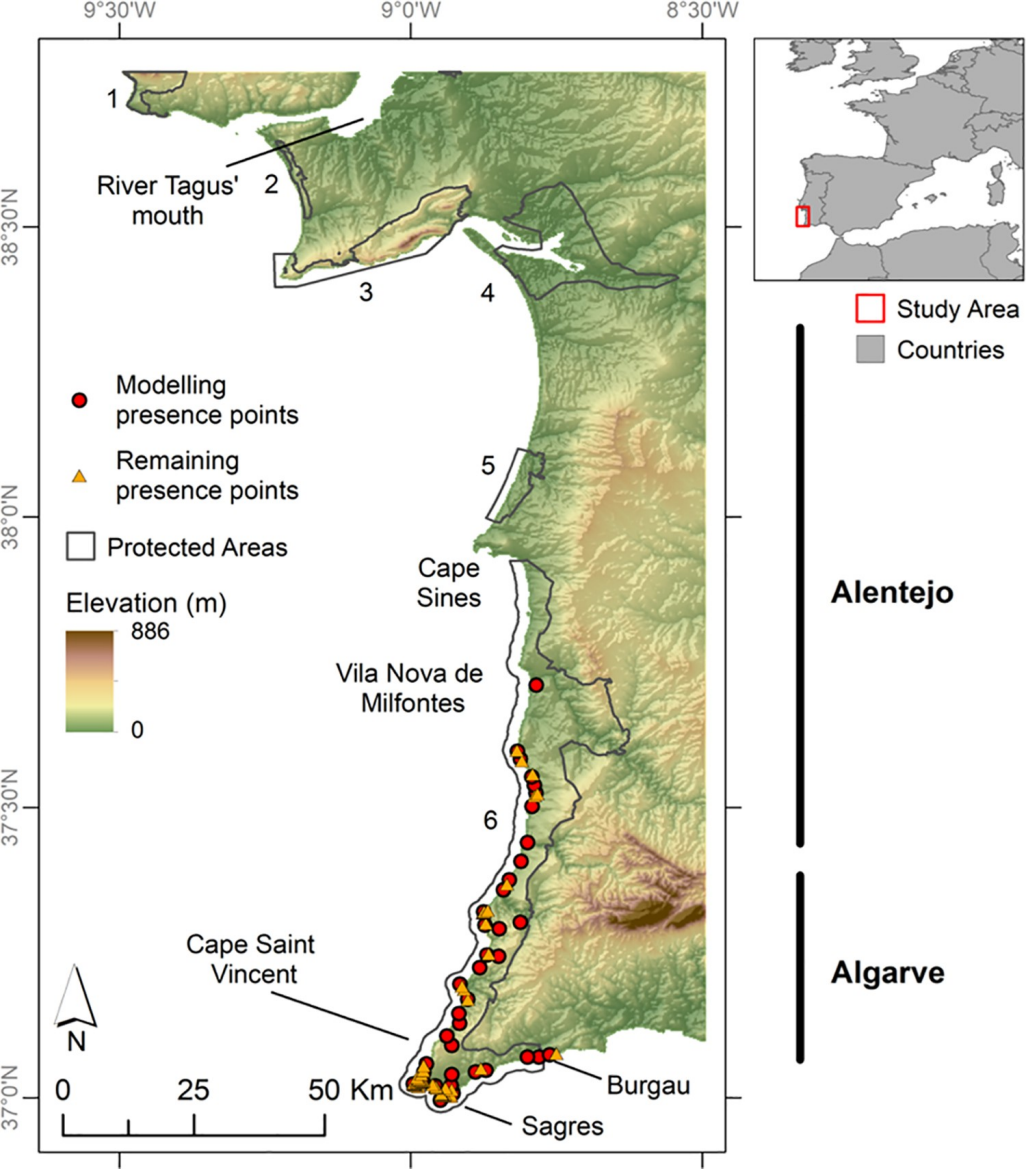
Location of the study area and spatial representation of the presence dataset. The 38 post-thinning presence points of *C*. *ladanifer* subsp. *sulcatus*, which were used to produce the ENM and its future projections, and the remaining 64 presence points, over an elevation layer [23]. Protected areas [24] are displayed as follows: 1 –Sintra-Cascais Natural Park; 2 –Costa da Caparica Fossil Cliff Protected Landscape; 3 –Arrábida Natural Park; 4 –Sado Estuary Natural Reserve; 5 –Santo André and Sancha Lagoons Natural Reserve; 6 –Southwest Alentejo and Vicentine Coast Natural Park. Other regions, mentioned in the text, are outlined. The top right inset displays the geographical location of the study area on a European and North African frame [25]. This figure was assembled using ArcGIS Desktop v10.8.1 [26].

The study area consisted in the Portuguese occidental southern region, embodying the known distribution of *C*. *ladanifer* subsp. *sulcatus* (Fig 1). The study area’s northernmost limit was the river Tagus’ mouth to account for future dispersal events towards this area. The eastern limit took into consideration a minimal dispersion along the Mediterranean Algarve coast observed in preliminary ENM projections. Besides, this is the most touristic region in the country, with the coastal landscape being dominated by buildings, thus leaving little space for plant populations to flourish, a situation hardly changeable in future decades.

### Presence data

The presence dataset was gathered by searching for “*Cistus ladanifer*” and “*Cistus palhinhae*” (synonym of *Cistus ladanifer* subsp. *sulcatus*) in the databases of GBIF [27–29], and iNaturalist [30]. All the corresponding 154 records were downloaded and cleaned, discarding those included in the following categories: (i) occurring far away from the known range of *C*. *ladanifer* subsp. *sulcatus* [31]; (ii) lacking expert-based confirmation, thus with dubious taxonomic identification of *C*. *ladanifer* subsp. *sulcatus*; or (iii) having an inaccuracy higher than 1 km, the spatial resolution used in this study (otherwise they could be assigned to the wrong grid cells [32]). The dataset was completed with 2 records from Quintela-Sabarís et al. [10] and 54 records provided by the Portuguese Society of Botany (http://www.spbotanica.pt/). This plant was taken as present near Peniche (Central coast of Portugal [31];) but this record was discarded for being uncertain (Miguel Porto, pers. comm.). All records were referenced to the World Geodetic System 1984, the coordinate system used throughout this work.

This 102 occurrences’ dataset (Fig 1 and S1 Table) included, however, spatially clustered data. This is a frequent drawback of opportunistic assemblages of presence records [14, 33, 34], which tend to be biased by geographical or environmental factors [35, 36] thus contributing to ENM overfitting [37, 38]. To overcome this issue, the package ‘spThin’ v0.2.0 [36] in R v4.0.2 [39] was used to filter the dataset with a distance of 1.5 km, which was found to be a good compromise between clustering reduction and the elimination of records. To guarantee that the maximum number of records distancing at least 1.5 km from each other were kept, spThin was run with 100 iterations, resulting in 38 non-clustered records, the final presence data used to produce the models (Fig 1).

### Environmental data

A set of 20 climatic and habitat-related variables was gathered and cropped using the study area’s limits (S2 Table). The bioclimatic variables were downloaded from WorldClim v1.4 [40] with a resolution of 30 arc-seconds (approximately 1 km). The Cliff Feature Delineation Tool and Baseline Builder [41], available for the same software, were employed to obtain an accurate characterization of southern Portugal’s coastal cliffs, resorting to the high spatial resolution Digital Terrain Model (2 x 2 m) created from airborne LiDAR data and provided by the Portuguese General Directorate of the Territory (https://www.dgterritorio.gov.pt/). Transects spaced 5 meters apart were defined (Fig in S1 Appendix) and, along each, points were obtained detailing information like their distance to the shoreline and altitude. The “Top” and “Toe” points (Fig in S1 Appendix)–the cliff’s top and base, respectively–were used to compute the slope. All the coastal regions with a slope value of at least 70% and whose “Top” point was located at no less than 15 meters of altitude were considered cliffs. This process was completed by visually inspecting the resulting “Top” cliff points in Google Earth [42] and editing them in ArcGIS Pro v2.7.0 [43] (see S1 Appendix for further information). The final 30 arc-seconds environmental variable resulted from the Euclidean distance in meters to the final set of “Top” points, computed in ArcGIS Desktop v10.8.1 [26].

Four uncorrelated continuous variables that revealed some contribution to model *C*. *ladanife*r subsp. *sulcatus*’ distribution according to prior trials were selected (using the commonly employed index of Pearson [44]: Pearson correlation < 0.75): Bio 9 –Mean Temperature of Driest Quarter, Bio 12 –Annual Precipitation, Bio 15 –Precipitation Seasonality and Distance to Cliffs (Table 1).

**Table 1:** Values of the selected environmental variables.

Variables	Present	Future						
		Year	RCP	ACCESS1-0	BCC-CSM1-1	CCSM4	MIROC-ESM	MRI-CGCM3
Bio 9: Mean Temperature of Driest Quarter (°C)	21.69 ± 0.72	2050	4.5	23.85 ± 0.84	23.85 ± 0.78	23.34 ± 0.84	25.16 ± 0.85	23.05 ± 0.79
				(19.9–25.3)	(19.9–25.1)	(19.5–24.9)	(21.0–26.8)	(19.1–24.3)
		2050	8.5	24.3 ± 0.89	24.42 ± 0.8	23.68 ± 0.92	26.04 ± 0.83	23.43 ± 0.84
				(20.3–25.8)	(20.4–25.7)	(19.5–25.2)	(22.0–27.4)	(19.5–24.8)
	(17.8–22.8)	2070	4.5	24.43 ± 0.87	23.98 ± 0.78	23.3 ± 0.86	26.44 ± 0.82	23.66 ± 0.79
				(20.5–25.9)	(20.0–25.2)	(19.2–24.7)	(22.5–27.8)	(19.7–24.9)
		2070	8.5	24.84 ± 0.96	25.76 ± 0.84	24.24 ± 1.0	27.46 ± 0.86	24.14 ± 0.88
				(20.6–26.6)	(21.7–27.1)	(20–25.9)	(23.4–28.9)	(20.1–25.6)
Bio 12: Annual Precipitation (mm)	60.01 ± 6.88	2050	4.5	48.77 ± 4.79	56.35 ± 6.36	49.94 ± 6.85	56.19 ± 6.23	64.68 ± 6.98
				(37.5–68.0)	(42.9–81.3)	(36.1–74.8)	(42.8–80.8)	(49.8–93.7)
		2050	8.5	46.15 ± 5.13	50.68 ± 6.61	46.88 ± 6.82	48.06 ± 5.58	69.68 ± 7.84
				(35.0–66.9)	(37.5–76.0)	(33.5–70.4)	(36.1–69.8)	(53.3–101.3)
	(45.8–87.3)	2070	4.5	49.65 ± 6.44	54.42 ± 6.59	48.24 ± 6.91	48.91 ± 6.04	61.90 ± 7.16
				(35.6–72.8)	(40.8–80.6)	(34.6–72.8)	(36.3–71.8)	(46.8–89.5)
		2070	8.5	42.91 ± 6.23	44.37 ± 5.69	45.41 ± 6.71	41.0 ± 5.6	63.97 ± 7.99
				(30.4–64.6)	(33.2–66.3)	(32.3–67.9)	(29.3–61.6)	(47.7–94.1)
Bio 15: Precipitation Seasonality (%)	64.73 ± 2.55	2050	4.5	80.84 ± 4.08	72.33 ± 2.77	69.37 ± 2.6	69.59 ± 2.72	72.04 ± 2.04
				(71.0–89.0)	(67.0–80.0)	(64.0–77.0)	(64.0–78.0)	(68.0–77.0)
		2050	8.5	78.49 ± 3.39	70.63 ± 1.84	71.08 ± 2.48	66.92 ± 2.56	79.26 ± 2.38
				(71.0–89.0)	(67.0–77.0)	(66.0–78.0)	(61.0–75.0)	(74.0–85.0)
	(60.0–72.0)							
		2070	4.5	74.29 ± 2.11	62.88 ± 1.95	69.93 ± 2.27	67.88 ± 2.87	71.78 ± 2.44
				(70.0–82.0)	(59.0–69.0)	(65.0–77.0)	(61.0–77.0)	(67.0–80.0)
		2070	8.5	96.35 ± 1.91	77.05 ± 1.59	73.66 ± 3.02	68.73 ± 2.0	85.56 ± 4.08
				(91.0–105.0)	(73.0–83.0)	(68.0–82.0)	(61.0–76.0)	(78.0–97.0)
Distance to Cliffs (m)	17358.91 ± 11491.13	-	-	-	-	-	-	-
	(0–48328.82)							

Average ± standard deviation (minimum–maximum) values of the four environmental variables selected from the total set of twenty variables (see S2 Table) for the study area. For the bioclimatic variables, both the values for present and future are displayed, for each general circulation model, period and Representative Concentration Pathway (RCP). The variable Distance to Cliffs was only available for the current time.

**Fig 2:**
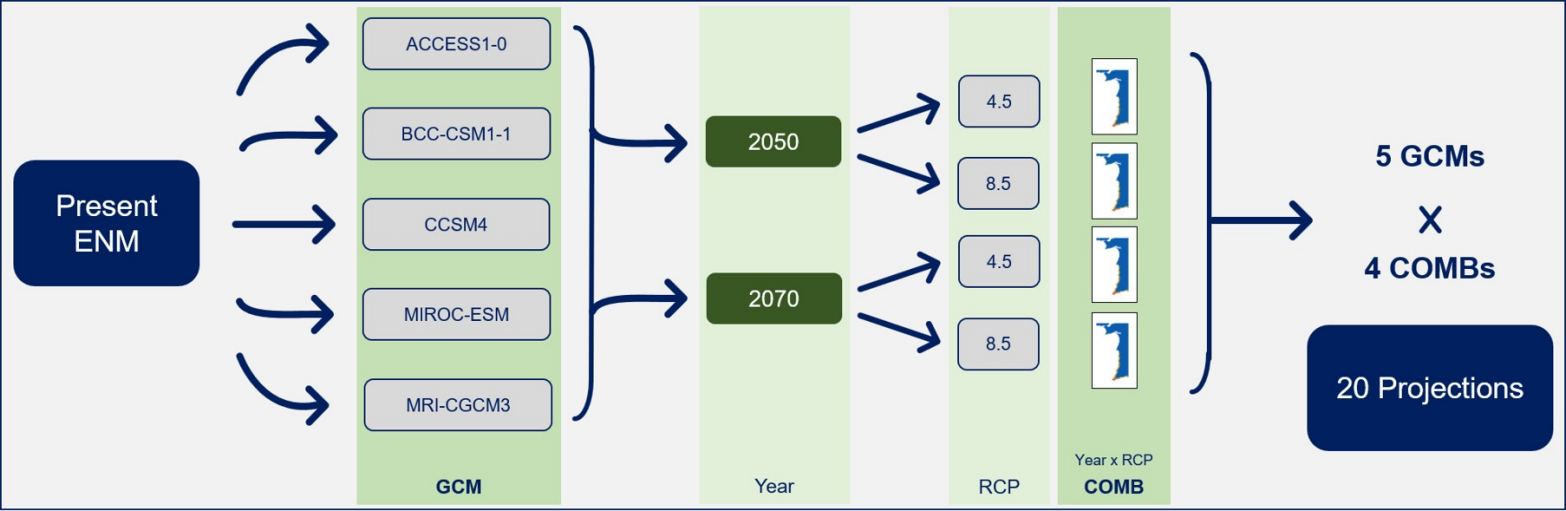
Methodological workflow for the projections of the Ecological Niche Model (ENM) into the future, for five General Circulation Models (GCMs). For each GCM, four combinations (COMBs) of years (2050 and 2070) and Representation Concentration Pathways (RCPs; 4.5 and 8.5) were produced.

To project the model into the future, five GCMs (ACCESS1-0, BCC-CSM1-1, CCSM4, MIROC-ESM and MRI-CGCM3) resulting from the Phase 5 of the Coupled Model Intercomparison Project (CMIP5) were used. They were available for the studied periods– 2050 (average for 2041–2060) and 2070 (average for 2061–2080)–and were characterized by a reasonable variability for the study area when included in a pool of 13 GCMs in GCM compareR web application [45], which could help transmitting a comprehensive perspective. Resorting to the WorldClim database, the bioclimatic variables were downloaded with a 30 arc-seconds resolution for each GCM, period, and two representation concentration pathways (RCPs): 4.5 and 8.5, referring to a moderate and a more serious greenhouse gas scenario, respectively [46]. The variable Distance to Cliffs was maintained as in the present since it will probably remain unchanged in the future time-slice considered (Table 1). The inclusion of static variables in ENM projections may be an advantageous strategy as demonstrated elsewhere [47]. Considering the five GCMs and the four combinations of future periods and RCPs, in total, 20 future projections were produced (Fig 2).

### Ecological niche modelling

The ENM was computed employing the widely-used software Maxent v3.4.1 [48]. Known for generating robust presence-background models, even with low sample sizes, it frequently outperforms other modelling techniques [49–52]. Firstly, the R package ‘ENMEval’ v0.3.1 [53] was used to tune the model to the optimal level of complexity with different combinations of Maxent’s feature classes (transformations of the predictor variables) and regularization multipliers (which penalize over-complex models) [54, 55]. Selecting the ‘randomkfold’ partitioning method with 10 kfolds and using the ‘maxent.jar’ algorithm, all combinations comprising the feature classes Linear (L), Quadratic (Q), Hinge (H), Linear + Product (LP), LQ, LH, PQ, PH, QH, LPQ, LPH, LQH and PQH and the sequence of regularization multipliers from 1 to 5 with an interval of 0.2 (thus 21 different values) were tested. The selected combination of parameters was the one with the smallest value concerning the Akaike Information Criterion for small samples (AICc): feature classes LQ and regularization multiplier 1. Given the narrow study area and the small sample size, the cross-validation method with 10 replicates was employed. Five thousand background points were randomly created over the study area (50.7% of all 9860 grid cells contained a background point), thus representing adequately its environmental spectrum [54]. A maximum number of 1000 iterations was used and the remaining settings were left as default.

The ENM performance was assessed using the area under the receiver operating characteristic curve (AUC) [56] and the true skill statistic (TSS) [57] for the testing data. They were calculated for each cross-validation replication and the values were subsequently averaged. The threshold employed both in TSS computation and for converting the suitability map into a geographical matrix of presences and absences, was the 10-percentile training presence threshold. The presence and absence maps allow one to assess range changes over time, a process that followed the comparison of two scenarios: with and without dispersal limitation. For the former, we used the minimum convex polygon plus a buffer of 2 km. Since the furthest record from the shore is located at approximately 5 km away, and to account for the buffer, the polygon was cropped by a maximum shore distance of 7 kilometers (from here on, dispersal limitation polygon). In this scenario, the polygon was considered the area beyond which *C*. *ladanifer* subsp. *sulcatus* was unable to disperse. For both of them, ArcGIS Desktop was used to compare the number and proportion of map cells that were predicted to be maintained, gained and lost over time according to each GCM and RCP.

### Land use analysis

In order to infer the land use that occupies the area where *C*. *ladanifer* subsp. *sulcatus* occurs at present and will occur in the future, the binary maps resulting from the ENM were compared with land use maps corresponding to the same periods. For the present, the CORINE Land Cover [58] 2018 map was used. This map and the CORINE Land Cover 2006 map, both categorized in five classes (Water, Forest, Scrubland, Cropland, Urban areas) and converted to a raster format in ArcGIS Desktop, were used to predict the land use for 2050 and 2070. This was done through the Patch-generating Land Use Simulation model v1.25 [59] (see S2 Appendix for further information). Discriminating the two periods (2050 and 2070), the proportion of each land use category in the total area of occurrence according to each ENM prediction was computed.

## Results

### Current potential distribution area

**Fig 3:**
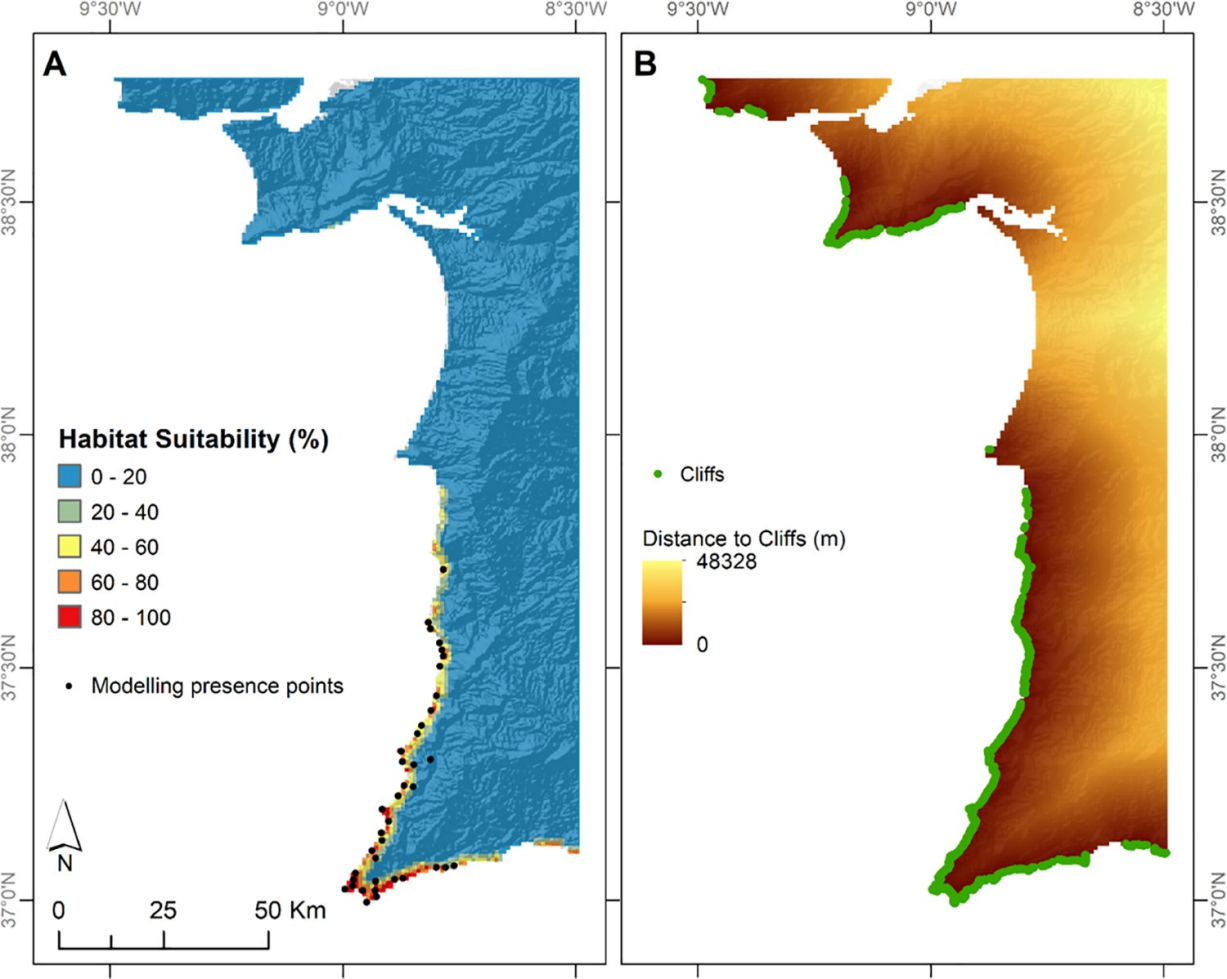
Habitat suitability map and coastal cliffs in the study area. (A) Current habitat suitability map and the 38 presence records used to produce it; (B) Identified cliffed coasts, and the distance between them and each location of the study area. This figure was assembled using ArcGIS Desktop v10.8.1 [26].

The current ecological niche of *Cistus ladanifer* subsp. *sulcatus* was concentrated in the coastal areas of south-western Portugal and the Cape Saint Vincent was found to be its hotspot (Fig 3A). The ENM was characterized by a high predictive accuracy: Test AUC = 0.98, which is close to the optimum AUC value of 1 [60]; Test TSS = 0.87, having a better performance than the reference value for defining a good model (0.6 [61]).

**Fig 4:**
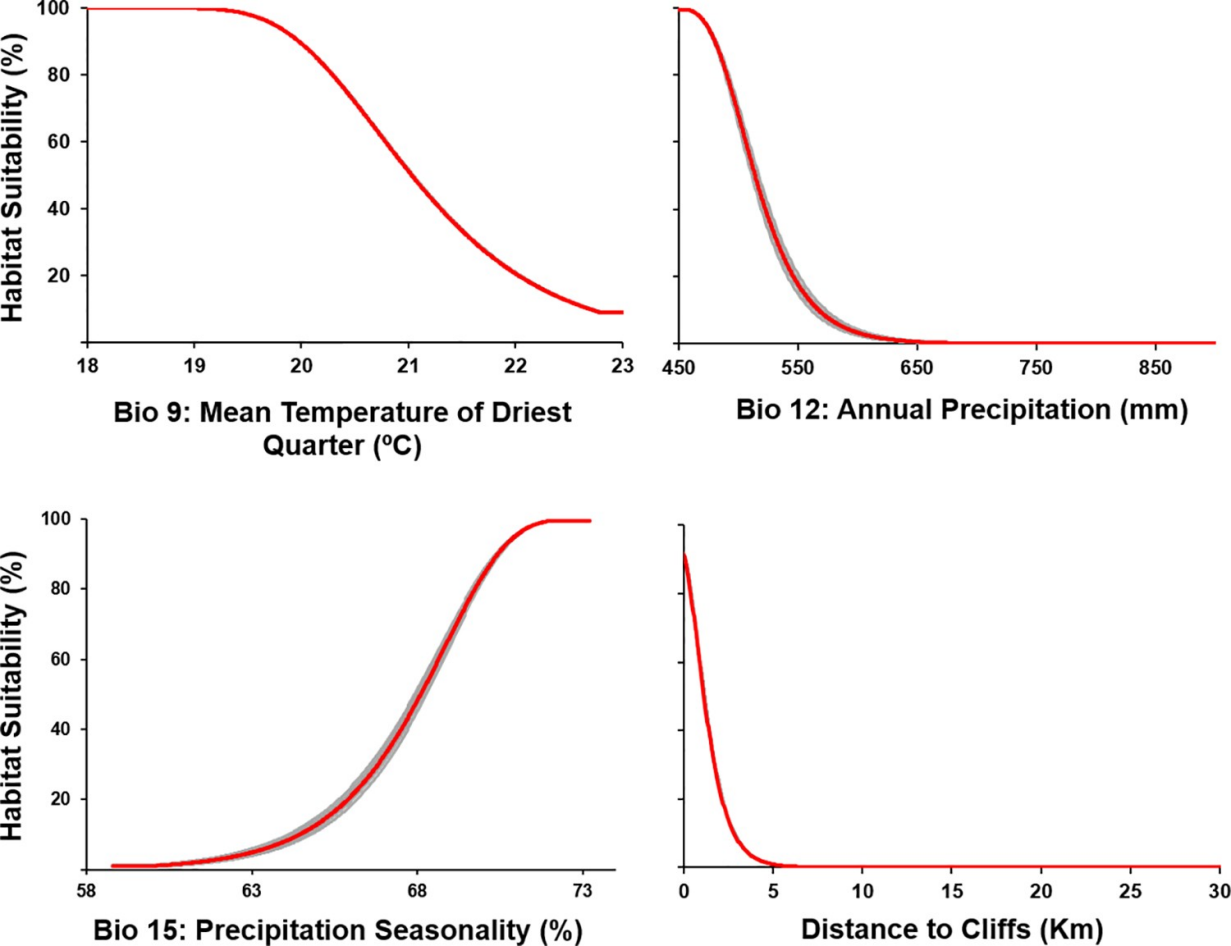
Response curves for each environmental variable. Plots resulting from the models created using only the corresponding variable. The grey colour represents cross-validation replications and the red colour represents the average of those ten replications.

The most relevant variable was the Distance to Cliffs, representing 81% of the average percentage contribution and ca. 92% of the average permutation importance (Table 2). Annual Precipitation had an average contribution of 13%, a value that was lower than 3% for both the Mean Temperature of Driest Quarter and the Precipitation Seasonality. However, at least one ENM replicate disclosed a percentage contribution of approximately 8%. Permutation importance was consistently low across bioclimatic variables (Table 2). Corroborating the identical visual pattern between occurrence records and cliffs (Fig 3), Maxent’s output unveiled a clear habitat suitability decrease with increasing distances to cliffs (Fig 4). The variables Mean Temperature of Driest Quarter and Annual Precipitation had similar patterns, though with less pronounced curves. On the other hand, suitability tended to increase with the Precipitation Seasonality.

**Table 2:** Average (minimum—maximum) percentage contribution and permutation importance of each environmental variable to the ecological niche model.

Variable	Percentage Contribution (%)	Permutation Importance (%)
Distance to Cliffs	81.38 (79.63–83.05)	92.17 (91.03–93.35)
Bio 12: Annual Precipitation	13.13 (9.16–15.42)	6.12 (5.41–6.97)
Bio 9: Mean Temperature of Driest Quarter	2.76 (1.75–3.47)	0.67 (0.29–0.96)
Bio 15: Precipitation Seasonality	2.73 (0.73–7.85)	1.04 (0.73–1.28)

### Future evolution of the geographical range

**Fig 5:**
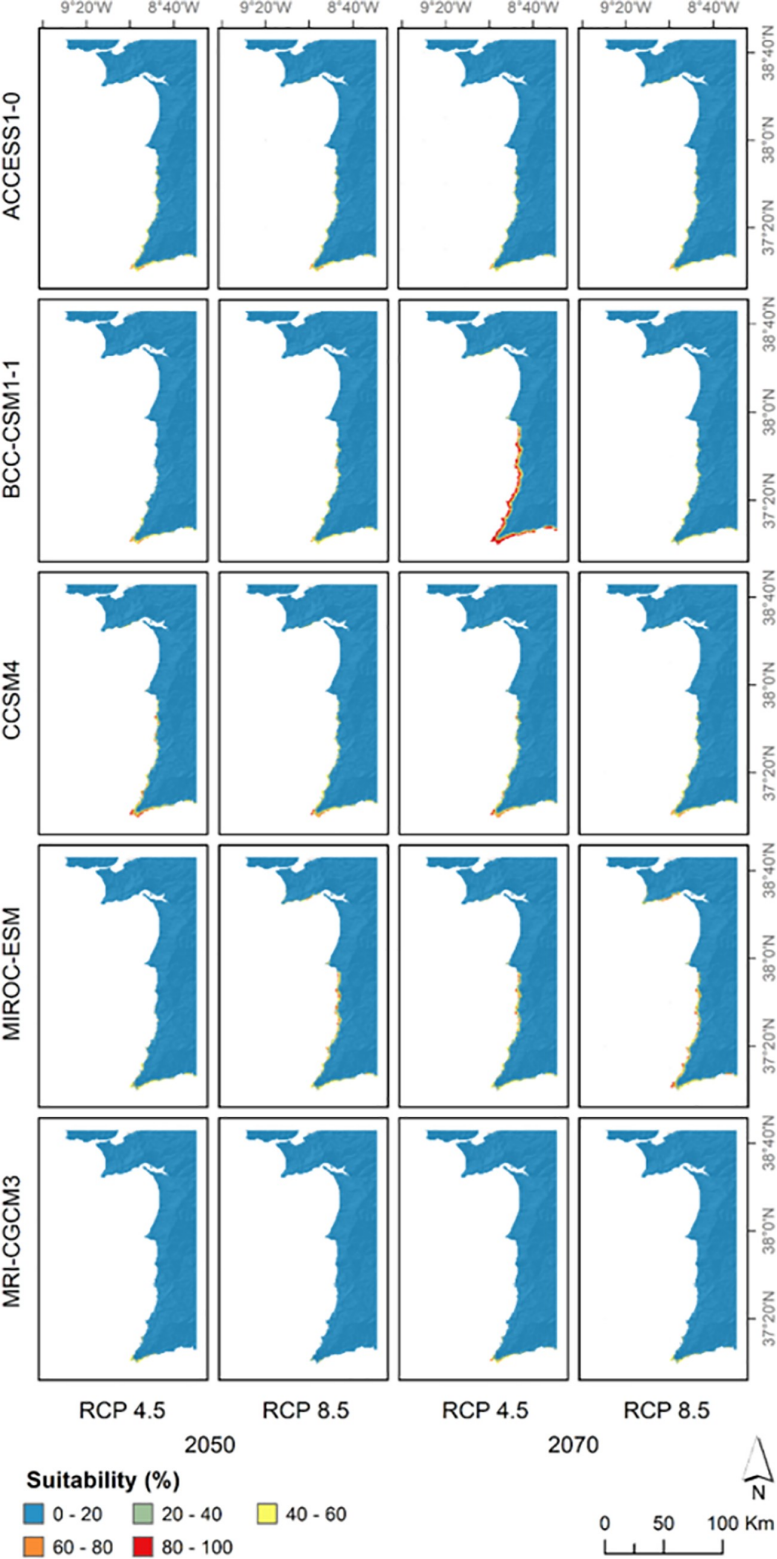
Predicted habitat suitability for the different future scenarios. Results are displayed in a matrix with lines corresponding to general circulation models and each column consisting of a future period and Representative Concentration Pathway (RCP) combination. This figure was assembled using ArcGIS Desktop v10.8.1 [26].

Most projections into the future suggested that suitable areas for *C*. *ladanifer* subsp. *sulcatus* will decrease in the future. Only the GCM BCC-SM1-1 had a projection (2070, RCP 4.5) showing the opposite trend, even though suitable areas remained concentrated along the coastline south of Cape Sines. In general, this was the most suitable area, followed by the Arrábida Natural Park in some cases (Fig 5). Equivalently to the present, the Cape Saint Vincent was selected as the most suitable area and was the only region where the plant will be able to persist according to the GCM MRI-GCGM3 projections. The only exception was verified for two scenarios of the GCM MIROC-ESM (2050, RCP 8.5; and 2070, RCP 4.5), where habitat suitability was low in Cape Saint Vincent and tended to increase further north.

**Fig 6:**
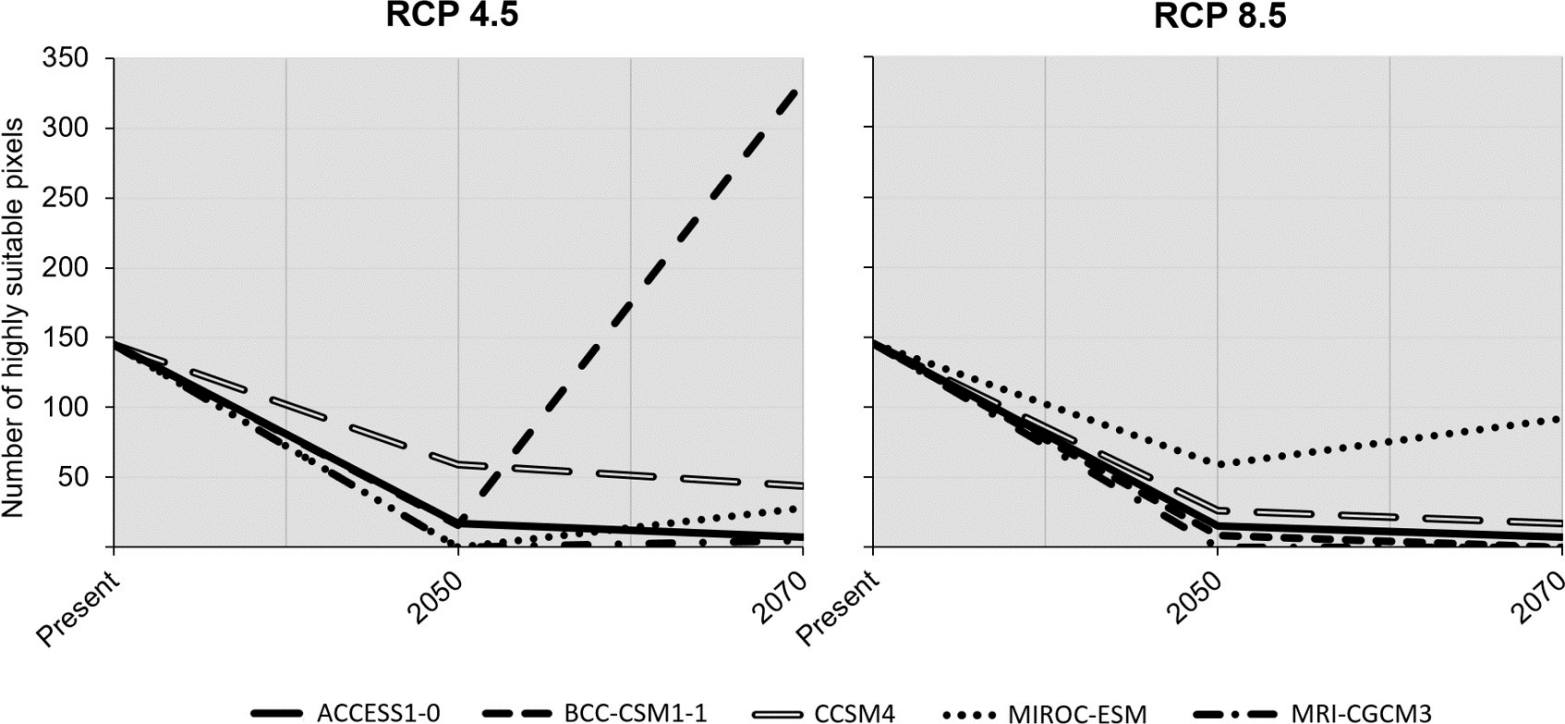
Extension of the most suitable area over time. Number of cells with suitability values above 60% over time (based on Figs 3 and 5), for Representation Concentration Pathways (RCPs) 4.5 and 8.5.

Overall, the number of cells with habitat suitability percentage values above 60% decreased considerably over time (Fig 6). Starting with 145 cells in this category at present, in most cases, they will be less than 50 in the future. The most preeminent deviation from this pattern was BCC-CSM1-1, which for the RCP 4.5 has more than doubled the number of high suitability cells by 2070, despite suffering an enormous reduction in 2050. The RCP 8.5 had the worst scenarios for the plant, with most GCMs not going beyond the 20 cells with a habitat suitability above 60%, especially in 2070. The exception was MIROC-ESM, that, despite the reduction of these cells compared to present, did not show dramatic losses (86 cells in 2050 and 53 cells in 2070; Fig 6).

**Fig 7:**
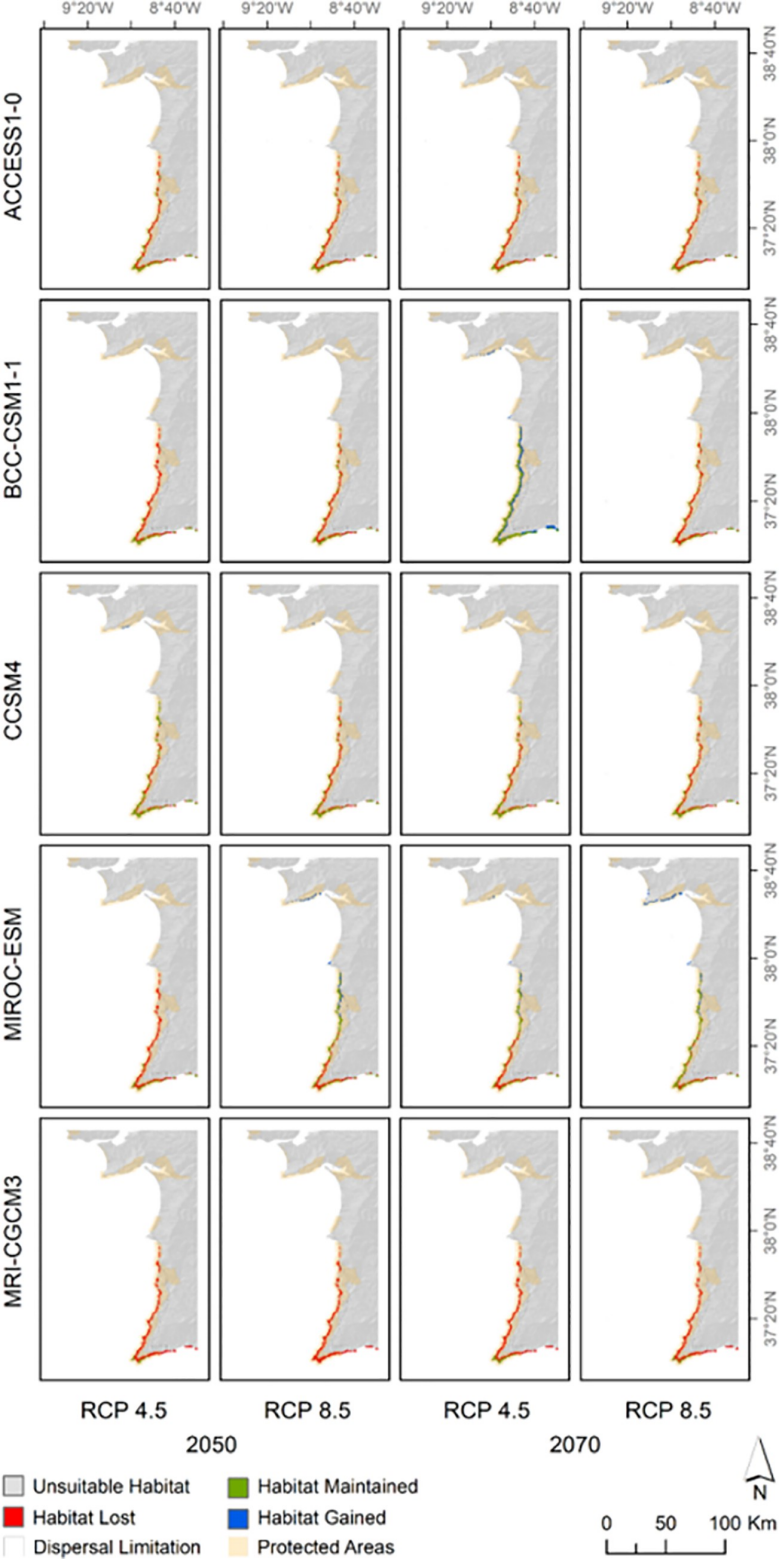
Predicted changes in suitable habitat for the different scenarios. Results are displayed in a matrix with lines corresponding to general circulation models and each column consisting of a future period and RCP (Representative Concentration Pathway) combination. Protected areas [24] and the dispersal limitation polygon are also displayed. This figure was assembled using ArcGIS Desktop v10.8.1 [26].

**Fig 8:**
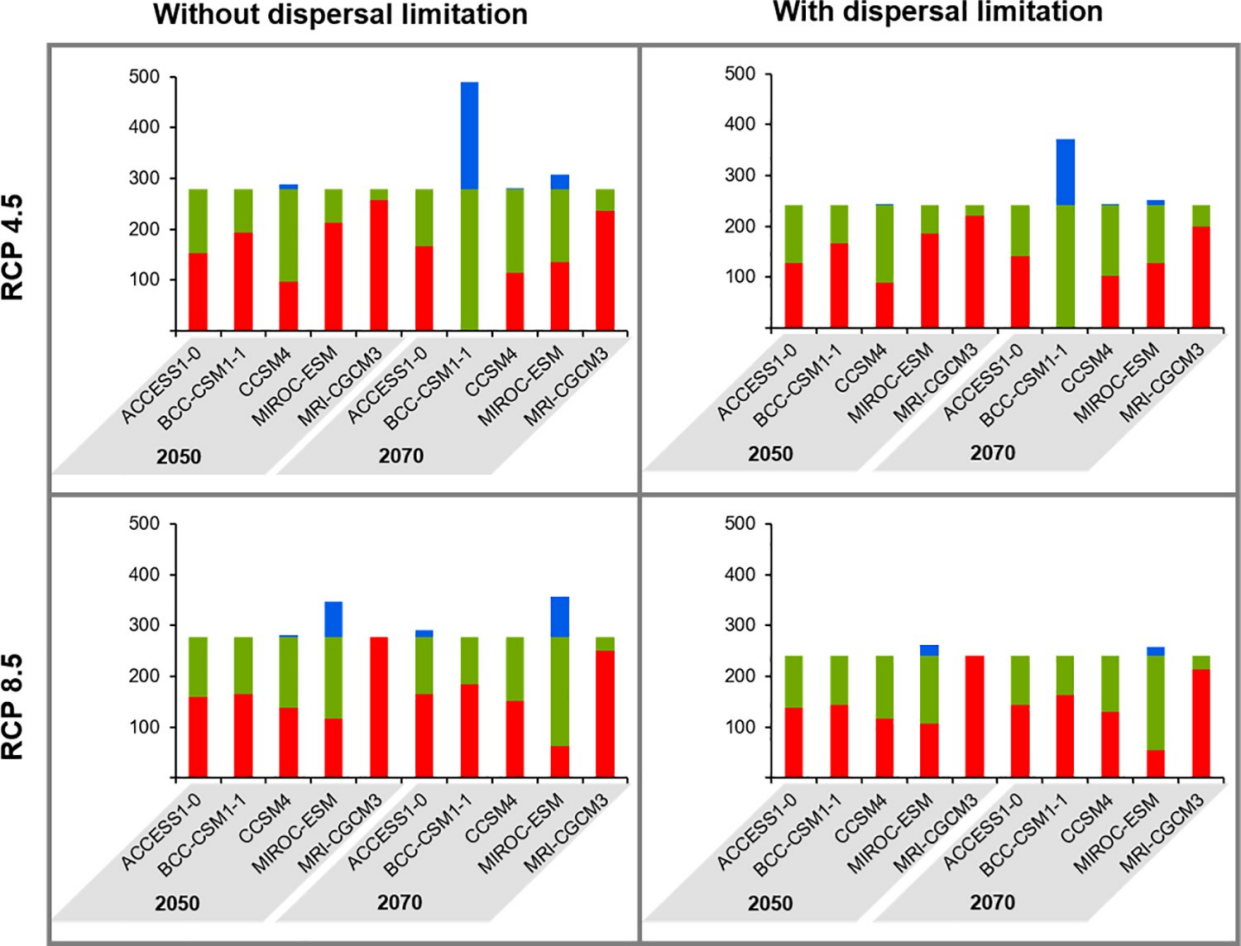
Number of cells in each category of predicted change. The colours correspond to the categories of habitat change as follows: Green–Habitat Maintained; Red–Habitat Lost; Blue–Habitat Gained. The bar plots on the left correspond to the unrestricted scenario, where *C*. *ladanifer* subsp. *sulcatus* can disperse everywhere, contrarily to the bar plots on the right, which consider the dispersal limitation polygon, represented in Fig 7, as the exclusive area where it may be present, both currently and in the future. The top bar plots illustrate the projections for the Representative Concentration Pathway (RCP) 4.5 while the bottom bar plots represent the RCP 8.5 projections.

The 10-percentile threshold of 0.3521 was applied to obtain maps discriminating suitable and unsuitable habitats, which were interpreted as presence-absence maps. Combining the current map with the 20 future projections (Fig 7), two general patterns were observed: (i) habitat loss was more prevalent in areas further from the coast; and (ii) habitat gain was much rarer, but when it happened it was predominant in both the strip between the northern half of *C*. *ladanifer* subsp. *sulcatus* distribution and Cape Sines and the Arrábida Natural Park. Most GCMs showed congruent projections, with substantial habitat loss and moderate levels of habitat maintenance, but there were remarkable exceptions. Despite following that general pattern, BCC-CSM1-1 predicted no habitat loss for 2070 and RCP 4.5, but instead an increase of 75.7% in the suitable area (Fig 8 and S3 Table). The MIROC-ESM scenarios also obtained contrasting results: whereas habitat loss was higher than 70% for 2050 and RCP 4.5, there was a slight habitat gain (5.7%) by 2070 according to the RCP 8.5. For the remaining scenarios (2050, RCP 8.5 and 2070, RCP 4.5), MIROC-ESM was also distinctive since habitat loss was more frequent in the southern part of the Vicentine coast rather than in latitudes further north. The GCM MRI-CGCM3 disclosed the worst scenarios, with habitat losses ranging between 84.3% and 100% in unconstrained scenarios (Fig 8 and S3 Table). The dispersal limitation polygon circumscribed the currently suitable area by 37 cells (13% of the 278 currently suitable cells), resulting in 241 cells. Compared with the opposite scenario, limiting the plant’s dispersal led frequently to the contraction of cells’ net gain percentages, suggesting that habitat gain was more common outside the dispersal limitation polygon. This limitation even converted the abovementioned habitat gain of 5.7% for MIROC-ESM in the scenario 2070 + RCP 8.5 into a 16% habitat loss (Fig 8 and S3 Table).

### Land use analysis

Scrublands were the most common category encompassing both present and future distribution predictions (Table 3). Cropland and Urban areas were the categories that followed, with the former being systematically the most relevant of the two except for all MRI-CGCM3 scenarios that did not predict the extinction in the future and for MIROC-ESM in 2050 and RCP 4.5. Water and Forest cells were practically insignificant in all scenarios and absent in 19 and 6 of them, respectively.

**Table 3:** Percentage of each land use category within the predicted area of occurrence according to each scenario.

Year	RCP	GCM	Water	Forest	Scrubland	Cropland	Urban areas
Present	-	-	0.41	2.07	54.55	35.95	7.02
2050	4.5	ACCESS1-0	0	1.77	63.72	24.78	9.73
		BCC-CSM1-1	0	1.35	67.57	16.22	14.86
		CCSM4	0	1.95	59.74	29.87	8.44
		MIROC-ESM	0	0	69.09	14.55	16.36
		MRI-CGCM3	0	0	66.67	9.52	23.81
	8.5	ACCESS1-0	0	0.96	62.50	25.96	10.58
		BCC-CSM1-1	0	1.02	62.24	26.53	10.20
		CCSM4	0	1.60	64.80	24.00	9.60
		MIROC-ESM	0	1.94	47.74	44.52	5.81
		MRI-CGCM3	0	0	0	0	0
2070	4.5	ACCESS1-0	0	1.01	61.62	27.27	10.10
		BCC-CSM1-1	0.54	1.61	47.45	44.50	5.90
		CCSM4	0	2.16	60.43	27.34	10.07
		MIROC-ESM	0	0.81	50.00	42.74	6.45
		MRI-CGCM3	0	0	66.67	14.29	19.05
	8.5	ACCESS1-0	0	1.02	61.22	27.55	10.20
		BCC-CSM1-1	0	0	61.54	30.77	7.69
		CCSM4	0	0.90	63.06	26.13	9.91
		MIROC-ESM	0	1.97	54.68	36.45	6.90
		MRI-CGCM3	0	0	70.37	7.41	22.22

## Discussion

### Climate change impacts

When projecting future range changes, uncertainty is inevitable and may arise from different sources, such as GCMs and RCPs [62, 63]. The current study unfolded contradicting results, with some scenarios suggesting an increase of the suitable area for *C*. *ladanifer* subsp. *sulcatus* but most pointing to its contraction. The MRI-CGCM3 stood out for predicting a worrying loss of all or nearly all suitable habitat. It was the GCM with the highest levels of annual precipitation, being quite distinct from the other four, particularly concerning the RCP 8.5. Excessive precipitation values in the future will likely have negative impacts on this subspecies, which is in line with its succulent-like ecophysiological strategy [21], helpful considering the extreme living conditions it faces, like strong winds and constant salt spray. Nonetheless, the results suggested that precipitation was relevant, especially if taking place at the appropriate season, which is not surprising given the highly marked rainy winters of the Mediterranean climate. If there was not a disproportionate contribution of the variable Distance to Cliffs, Precipitation Seasonality would probably be recognized as a much more influential variable, as it is common in Mediterranean species [64].

Remarkably, for most GCMs, the projections considering the most severe greenhouse gas scenario (RCP 8.5) were identical to the smoother RCP 4.5. They even achieved quite favourable results for MIROC-ESM and, in 2050, for BCC-CSM1-1. Similarly, the climatic conditions in 2070 had no magnified impacts, resulting in predictions consistent with those of 2050. The next decades could thus entail the major challenges for this plant, leading to a sharp decrease in suitable areas. Higher temperatures and lower precipitation levels beyond 2050 appear insufficient to exacerbate such an effect. This is likely due to the well-known drought resistance mechanisms of *C*. *ladanifer* [65–67], resembling those making some taxa particularly resilient to aridity [68]. Nevertheless, as the rate of this plant’s response to climatic alterations is not known, if a substantial contraction is verified by 2050 (as predicted by BCC-CSM1-1 and MIROC-ESM), it remains uncertain how fast it will regain the habitat that was lost. Habitat suitability can fluctuate in such a way that all study area is considered unsuitable by 2050, but in 2070 some cells return to being suitable (MRI-CGCM3). This emphasizes the need for an ecological debate over ENM results [69] which, in the present study, were obtained relying simply on machine-learning computations. If the whole study area turns into unsuitable habitat and the taxon becomes extinct by 2050, no matter the extent of suitable area in a distant future, it will no longer be occupied.

### Range limiting factors in the future

Climate will not play an exclusive role in whatever changes taking place. Meeting initial expectations, cliffed coasts were demonstrated to be fundamental for this subspecies given the sharp decrease in habitat suitability with increasing distances to these wave-eroded formations. If this variable was not included, the ENM would likely predict the presence of *C*. *ladanifer* subsp. *sulcatus* far inland, as verified for other coastal plants whose niche was modelled using only bioclimatic variables [70, 71]. In a first approach, other non-climatic variables were considered, namely the Edaphic Composition of the study area, whose importance is well known for this taxon [13] and in general for the Iberian flora [72, 73], but its contribution to the ENM was minimal. Possibly, larger sample sizes or finer spatial resolutions could bring different results. Land use could also be included as an ENM variable, but then results would no longer be interpreted as the plant’s ecological niche due to the anthropogenic influence. Land use was thus considered in a subsequent analysis, as discussed below.

If terrestrial taxa occurring exclusively along the shore have their fundamental niches highly restricted, such circumstance is further heightened in those that are dependent on a specific coast type. The results that were presented above suggest that the only area including suitable habitats outside the Vicentine coast is the Arrábida Natural Park, although these coastal cliffs are roughly 90 km away from the northernmost confirmed record of *C*. *ladanifer* subsp. *sulcatus*. In the literature, no information is currently available concerning this subspecies’ dispersal ability, contrarily to *C*. *ladanifer subsp*. *ladanifer*. Regarding the latter, Bastida and Talavera [74] showed that, on average, only 1.6% of seeds are dispersed over a minimal distance of 40 cm from the mother plant. Ants like *Goniomma kugleri* Espadaler [75] can increase dispersion by a few meters (as documented for another Mediterranean plant-ant interaction [76]) and the red deer (*Cervus elaphus* L.) is able to spread germinable seeds over much larger distances [77]. With this ungulate absent from the current range of *C*. *ladanifer* subsp. *sulcatus*, *G*. *kugleri* and other ants are likely to be the main dispersion vector.

Despite the absence of long distance dispersal mechanisms, *C*. *ladanifer* was able to cross the Strait of Gibraltar [9] and a similar event could be hypothesized for the *sulcatus* subspecies. However, that dispersal took hundreds of thousands of years and was only possible due to the widespread availability and continuity of suitable habitat, assumptions unverified for *C*. *ladanifer* subsp. *sulcatus*. Consequently, it is unlikely that the Arrábida Natural Park will be reachable for this plant over the next decades. Among the areas determined as suitable along the coasts of Alentejo and Algarve, those north to Vila Nova de Milfontes and east to Burgau lack confirmed records, suggesting a limited range filling [78]. This evidence could be a consequence of (i) biotic interactions like competition (which this plant tends to avoid [13]), (ii) abiotic factors not explored here, or (iii) extremely slow dispersal rates. In summary, there is a clear tendency for this subspecies to remain confined to the south-western edge of Europe and cope with climate change there.

The present work reinforces the need to examine species’ ecological attributes when designing ENMs. This is specifically relevant for specialist and narrowly endemic taxa. Besides the decisive relevance of littoral cliffs on *C*. *ladanifer* subsp. *sulcatus*’ distribution, accounting not for dispersal limitations would lead to potentially misleading conclusions, which should be avoided whenever possible in conservation research [79].

### Conservation implications

The methodology used in the present study addressed solely the responses of *C*. *ladanifer* subsp. *sulcatus* to environmental factors. Therefore, results should be acknowledged with caution as human pressure and other habitat disturbances may further constrain its niche. Although the Southwest Alentejo and Vicentine Coast Natural Park is a protected area and part of the Natura 2000 European Network, it includes several invasive species, like the ice plant *Carpobrotus edulis* (L.) N. E. Br. or the capeweed *Arctotheca calendula* (L.) Levyns, which, if not detected in an early stage [80], may pose relevant threats to native plants, especially microendemic taxa.

This Park is fairly unurbanized and revealed no considerable expansion of urban areas between 2006 and 2018, a pattern that will likely be reproduced in the future (Fig in S2 Appendix). The exception was Sagres, where *C*. *ladanifer* subsp. *sulcatus* is particularly abundant. This region explains the percentage values around 20% for urban areas within the extremely reduced predicted distribution concerning MRI-CGCM3 (Table 3). Even though urban cells may include patches with suitable habitat for this plant, human presence could lead to menacing activities such as the illegal use of vehicles and waste dumping in coastal heaths [13]. Therefore, in such eventualities, the worrying losses of habitat predicted by MRI-CGCM3 would be even greater. This could also occur in respect to cropland areas, which are common in the northern part of the Vicentine coast. In most scenarios, MIROC-ESM suggested that this will be the principal area where habitat will be maintained, but ecologically unsuitable land uses have the potential of further constraining it. Throughout this area, where *C*. *ladanifer* subsp. *sulcatus* populations tend to be more isolated, temporary crops and some scrublands have been progressively converted into other agricultural uses such as nursery agriculture. Even though croplands will likely decrease in the future across the study area, they will continue to dominate this region (Fig in S2 Appendix). Relevant human pressures also derive from tourism, which is particularly relevant during the high season and tends to be concentrated along the shore [81].

Impacts on this taxon will also affect the entire ecosystem it is included in. It is worth noting that the endemic phytoassociation *Genisto triacanthi-Cistetum palhinhae* Rivas-Martínez, Lousã, T.E. Díaz, Fernández-González & J.C. Costa [12], just as other unique typologies of southern Portugal, like the extremely threatened oak groves of *Quercus faginea* Lam. and *Q*. *canariensis* Wiild. or the riparian forests where the relic species *Rhododendron ponticum* (Boiss. & Reut.) Hand.-Mazz. occurs, are predicted to have their spatial representation reduced over time [82]. The Alentejo coast, where future range changes could be considerable [82], is a territory where we can find several endemic plants, including the “endangered” *Plantago almogravensis* Franco and *Herniaria algarvica* Chaudhri, and the “vulnerable” *Diplotaxis siifolia* (Welw. ex Samp.) Mart.-Laborde [83]. Even more restricted than *C*. *ladanifer* subsp. *sulcatus*, and forming with it the maritime heaths of Cape Sines and its vicinities, are for example *Astragalus tragacantha* L., *Silene rothmaleri* P. Silva, *Triplachne nitens* (Guss.) Link, *Helianthemum marifolium* subsp. *origanifolium* (Lam.) G.López and *Ulex erinaceus* Welw. ex Webb.

## Conclusions

Climate change is expected to reduce meaningfully the range of *C*. *ladanifer* subsp. *sulcatus* in the future, although more moderate scenarios could occur. This study confirmed the tight affinity between this plant and cliffed coasts, which are a pivotal condition for habitat suitability. Confined by long sands in the north and by urban landscapes in the southeast, where the Atlantic influence is replaced by a quiet Mediterranean Sea, no considerable expansion events are anticipated. Along these lines, population trends for this unique lineage of *C*. *ladanifer* should be followed up carefully in order to improve conservation policies aimed at protecting this subspecies and taxa co-occurring in the Vicentine coast, one of the major hotspots of Iberian plant diversity and endemism.

## Supporting information

S1 Appendix Detailed generation of the variable “Distance to Cliffs”. (PDF)

S2 Appendix Simulation of future land use layers. (PDF)

S1 Table Presence data. Total set of 102 distribution records, 38 of which selected to be used as input in the Ecological Niche Model.
(PDF)

S2 Table Environmental variables. Total set of environmental variables gathered for the present study, their units, mean ± standard deviation (minimum—maximum) values for the study area, and the source to which we resorted to obtain them.
(PDF)

S3 Table Summary of predicted changes in habitat suitability for each future prediction. The current number of suitable cells is compared with the future number of suitable cells and the resulting net gain values and percentages are presented. This comparison is made for the unrestricted scenario where *C*. *ladanifer* subsp. *sulcatus* can disperse everywhere, contrarily to the scenario with dispersal limitation, which considers the dispersal limitation polygon, represented in Fig 7, as the exclusive area where it may be present, both currently and in the future. RCP–Representation Concentration Pathways; GCM–General Circulation Models.
(PDF)
