# Supplementary material for: The role of littoral cliffs in the niche delimitation on a microendemic plant facing climate change
Source: PLoS One. 2021 Oct 22;16(10):e0258976. doi: 10.1371/journal.pone.0258976 (PMC8535191; doi:10.1371/journal.pone.0258976)
Supplement: S1 Appendix — (PDF) [file pone.0258976.s001.pdf]

The environmental variable Distance to Cliffs was computed resorting to a Digital Terrain Model (DTM) obtained for the Portuguese coast with the LiDAR technology in 2011 and provided to us by the Portuguese General Directory of the Territory (https://www.dgterritorio.gov.pt/). This layer had a resolution of 2 meters and was converted to a raster format in R v4.0.2 [1]. In order to shorten the computational processing effort, we divided this DTM into 18 different parts and administered the Baseline Builder and Cliff Feature Delineation Tools [2] in ArcGIS Desktop v10.8.1 [3] for each separately.

Using the default settings, the Baseline Builder tool automatically identified an offshore baseline, which was the base reference to delineate the coastal profile. However, as it was an automatic process, some baseline sections were produced in the wrong areas, requiring them to be edited in ArcGIS Desktop. Using this edited baseline as input, along with the DTM, the final results were obtained with the Cliff Feature Delineation Tool, applying the Refined Delineation mode with the High Pass method. The landward transect distance (i.e. the area each transect traverses, from the baseline towards land) was set to 200 meters and the remaining settings were set as default. The results included a "Top" and a "Toe" point for each transect, which were used to calculate the slope at the respective region, dividing the difference in altitude (altitude of "Top" point – altitude of "Toe" point) by the difference in the distance to the baseline (distance of "Top" point – distance of "Toe" point). We considered as cliffed coasts all the areas with a slope value of at least 70% and with a "Top" point with an altitude value of at least 15 meters. Using ArcGIS Pro v2.7.0 [4], we edited this output to eliminate the "Top" points that were wrongly assigned as cliffs. In the end, we computed the Euclidean distance to these points in ArcGIS Desktop, originating the 30 arc-seconds raster layer used as environmental variable.

S1 Appendix-Fig shows a portion of the study area with all the features that were mentioned above. Although the Cliff Feature Delineation Tool had wrongly generated some "Toe" points above the cliff base, leading to some incorrect cliff identifications, the resolution of the environmental variable (approximately 1 km) cancels out this effect given the vast amount of transects within each cell.

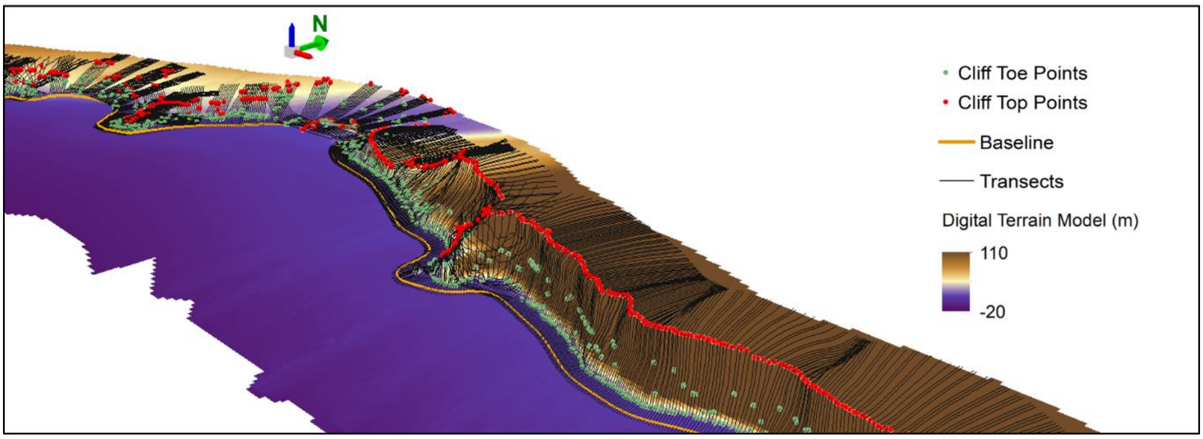

S1 Appendix-Fig. Digital Terrain Model and spatial features used to identify cliffed coasts. Portion of the study area, where there are displayed the Digital Terrain Model (https://www.dgterritorio.gov.pt/) and the Baseline that were used as inputs to produce the transects and their respective "Toe" and "Top" points. In the top left corner, the coast topography is flat, without cliffs, contrarily to the centre and bottom right corner of the image, where cliff profiles are easily deducted. This figure was assembled using ArcGIS Pro v2.7.0 [4].

References

1. R Core Team. R: A language and environment for statistical computing. Vienna, Austria: R Foundation for Statistical Computing; 2020. Available from: https://www.R-project.org/

2. Seymour AC, Hapke CJ, Warrick J. Cliff Feature Delineation Tool and Baseline Builder v1.0. US Geological Survey Software Release. 2020. doi: 10.5066/P9UKW7PO

3. ESRI. ArcGIS Desktop. Redlands, CA: Environmental Systems Research Institute; 2020.

4. ESRI. ArcGIS Pro. Redlands, CA: Environmental Systems Research Institute; 2020.
