# Supplementary material for: The role of littoral cliffs in the niche delimitation on a microendemic plant facing climate change
Source: PLoS One. 2021 Oct 22;16(10):e0258976. doi: 10.1371/journal.pone.0258976 (PMC8535191; doi:10.1371/journal.pone.0258976)
Supplement: S2 Appendix — (PDF) [file pone.0258976.s002.pdf]

The land use for 2006 and 2018 was obtained from CORINE Land Cover vectoral data [1], whose categories were grouped into 5 classes: Water, Forest, Scrubland, Cropland, Urban areas (Table A) in ArcGIS Desktop v10.8.1 [2]. The same software was employed to convert them to a 1-km raster format, the same resolution used throughout this work. Land use layers for 2006 and 2018 were used as a basis to simulate future land use maps with the Patch-generating Land Use Simulation model v1.25 [3]. This simulation model requires a set of relevant variables of different types, which were gathered from different sources (Table B). The Edaphic Composition was originally downloaded in a shapefile format and then converted to raster. The Gross Domestic Product and the Population size data were available in tables discriminating values for territorial units, whose shapefile was also obtained. A spatial join between these two types of information was performed in ArcGIS Desktop and the resulting layer was converted to raster. All variables representing distances to cities, infrastructures or water were computed in ArcGIS Desktop using shapefile data from multiple sources.

The simulation process was divided into 3 main parts: (i) Extract land expansion; (ii) Land expansion analysis strategy (LEAS); and (iii) Cellular Automata based on multiple random seeds (CARS). The first step compared the maps of 2006 and 2018 and identified the cells that had a category in 2006 and were replaced by another in 2018. The second step performed a random forest regression, using as input the land expansion map resulting from the first step and the assembled variables. Here, the number of regression trees was set to 10000 and a sampling rate of 0.1 was used. The mTry parameter was left as the default value (14). As a result, 5 raster layers (one for each land use category) were obtained, each detailing the development potential of each category across the study area. The third step used as a basis the land use map of 2018 and the 5 development potential layers. The parameters were set as follows: Neighbourhood size = 3, Patch generation threshold = 0.5, Expansion coefficient = 0.1; Percentage of seeds = 0.0001. The categories “Water” and “Urban areas” were set as immutable, since these are not expected to change to another land use. The neighbourhood weights were assigned according to the land expansion map obtained in the first step. The weight of each category was equal to the number of cells that changed to that category in 2018 divided by the total number of cells that changed in that year. The land demands, *i.e.* the number of cells of each category in the final simulation maps (which are different in 2050 and 2070), were obtained through a Markov chain prediction.

The CARS analysis was run again to assess the model accuracy. This was done by predicting the land use for 2018 and comparing it with the real map for this year. Starting with the land use map for 2006, all the parameters and the remaining input layers were maintained, except land demands, which were set to the number of cells of each land use category in the real land use map for 2018. The comparison between the real map and the simulation led to a Kappa coefficient of 0.841 and an overall accuracy of 0.885.

The resulting layers are depicted in S2 Appendix-Fig and the proportion of each land use category is detailed in Table C. The differences were small, especially for the Vicentine coast, but there was a slight increase of scrublands and urban areas counteracted by a decrease on croplands and forested areas.

S2 Appendix-Table A. Land use classification. The detailed classes in the original CORINE Land Cover layer [1] reclassified into the 5 classes used in this work.

Land Use	CORINE Land Cover class (Code)
Water	Inland marshes (411)
	Salt marshes (421)
	Salines (422)
	Intertidal flats (423)
	Water courses (511)
	Water bodies (512)
	Coastal lagoons (521)
	Estuaries (522)
Forest	Broad-leaved forest (311)
	Coniferous forest (312)
	Mixed forest (312)
Scrubland	Natural grasslands (321)
	Moors and heathland (322)
	Sclerophyllous vegetation (323)
	Transitional woodland-shrub (324)
	Beaches, dunes, sands (331)
	Bare rocks (332)
	Sparsely vegetated areas (333)
	Burnt areas (334)
Cropland	Non-irrigated arable land (211)
	Permanently irrigated land (212)
	Rice fields (213)
	Vineyards (221)
	Fruit trees and berry plantations (222)
	Olive groves (223)
	Pastures (231)
	Annual crops associated with permanent crops (241)
	Complex cultivation patterns (242)
	Land principally occupied by agriculture, with significant areas of natural vegetation (243)
	Agro-forestry areas (244)
Urban Areas	Continuous urban fabric (111)
	Discontinuous urban fabric (112)
	Industrial or commercial units (121)
	Road and rail networks and associated land (122)
	Port areas (123)
	Airports (124)
	Mineral extraction sites (131)
	Dump sites (132)
	Construction sites (133)
	Green urban areas (141)
	Sport and leisure facilities (142)

S2 Appendix-Table B. Spatial layers used to compute land use simulations for future periods. Total set of layers, their units, mean ± standard deviation (minimum – maximum) values (continuous variables) and classes (categorical variables) for the study area, and the source to which we resorted to obtain them.

Type	Name	Values (Mean ± Std. Dev. (Min. – Max.)) and Classes	Source
Land use	Land use 2006 (Classes)<br>Land use 2018 (Classes)	Water<br>Forest<br>Scrubland<br>Cropland<br>Urban Areas	CORINE Land Cover - Copernicus [1]
Land use Constraint	Permanent water (Classes)	Permanent water<br>Non-permanent water	European Environment Agency (https://www.eea.europa.eu/data-and-maps/)
Socioeconomic data	Population Size 2011	(30.89 ± 58.77)<br>(0 – 1033)	Calculated in ArcGIS Desktop v10.8.1 [2], using table and geographical (shapefile) data from the Portuguese National Institute of Statistics (https://www.ine.pt/)
	Distance to cities (m)	(18326 ± 11930)<br>(0 – 50624)	Calculated in ArcGIS Desktop v10.8.1 [2], using information from the Portuguese National Institute of Statistics (https://www.ine.pt/)
	Gross Domestic Product 2018 (€)	(23 M ± 31 M)<br>(2 M – 74 M)	Calculated in ArcGIS Desktop v10.8.1 [2], using table data from the Contemporaneous Portugal Database (https://www.pordata.pt/Municipios/PIB+(base+2016)-894), and geographical (shapefile) data from the Portuguese General Directorate of the Territory (https://www.dgterritorio.gov.pt/cartografia/cartografia-tematica/caop)
	Distance to highways (m)	(13063 ± 12199)<br>(0 – 48043)	Calculated in ArcGIS Desktop v10.8.1 [2], using information from OpenStreetMap (https://www.openstreetmap.org/)
	Distance to primary roads (m)	(2604 ± 2436)<br>(0 – 12456)	
	Distance to secondary roads (m)	(5056 ± 5636)<br>(0 – 35163)	
	Distance to tertiary roads (m)	(1784 ± 1659)<br>(0 – 9698)	
	Distance to residential roads (m)	(1257 ± 1254)<br>(0 – 7457)	
	Distance to railways (m)	(9561 ± 7944)<br>(0 – 33554)	
Climatic and environmental data	Edaphic Composition[a]	Litholic Soils<br>Poorly Unsaturated Clay Soils<br>Incipient Soils<br>Calcareous Soils<br>Reddish-Brown Clays<br>Rock Outcrops<br>Hydromorphic Soils<br>Brown Clays<br>Black Clays<br>Molic Soils<br>Halomorphic Soils<br>Podzolised Soils<br>Hydromorphic Organic Soils<br>Others	Portuguese General Directorate of Agriculture and Rural Development (https://www.dgadr.gov.pt/)
	Annual Mean Temperature (ºC)	(16.5 ± 4.97)<br>(12.4 – 17.2)	WorldClim v1.4 [4]
	Annual Precipitation (mm)	(49.50 ± 5.74)<br>(38 – 72)	
	Digital Terrain Model (m)	(95.15 ± 82.91)<br>(-1 – 825)	WorldClim v2.1 [5]
	Slope (%)	(1.74 ± 1.90)<br>(0 – 20.82)	Calculated in ArcGIS Desktop v10.8.1 [2], using the Digital Terrain Model from WorldClim v2.1 [5]
	Distance to water (m)	(2484 ± 2022)<br>(0 – 11863)	Calculated in ArcGIS Desktop v10.8.1 [3], using information from the European Environment Agency (https://www.eea.europa.eu/data-and-maps/data/wise-wfd-protected-areas-1/data-download)

[a] Originally, the Edaphic Composition variable had far more detailed categories, but to avoid that excessive complexity, they were grouped according to the general soil families occurring in the study area.

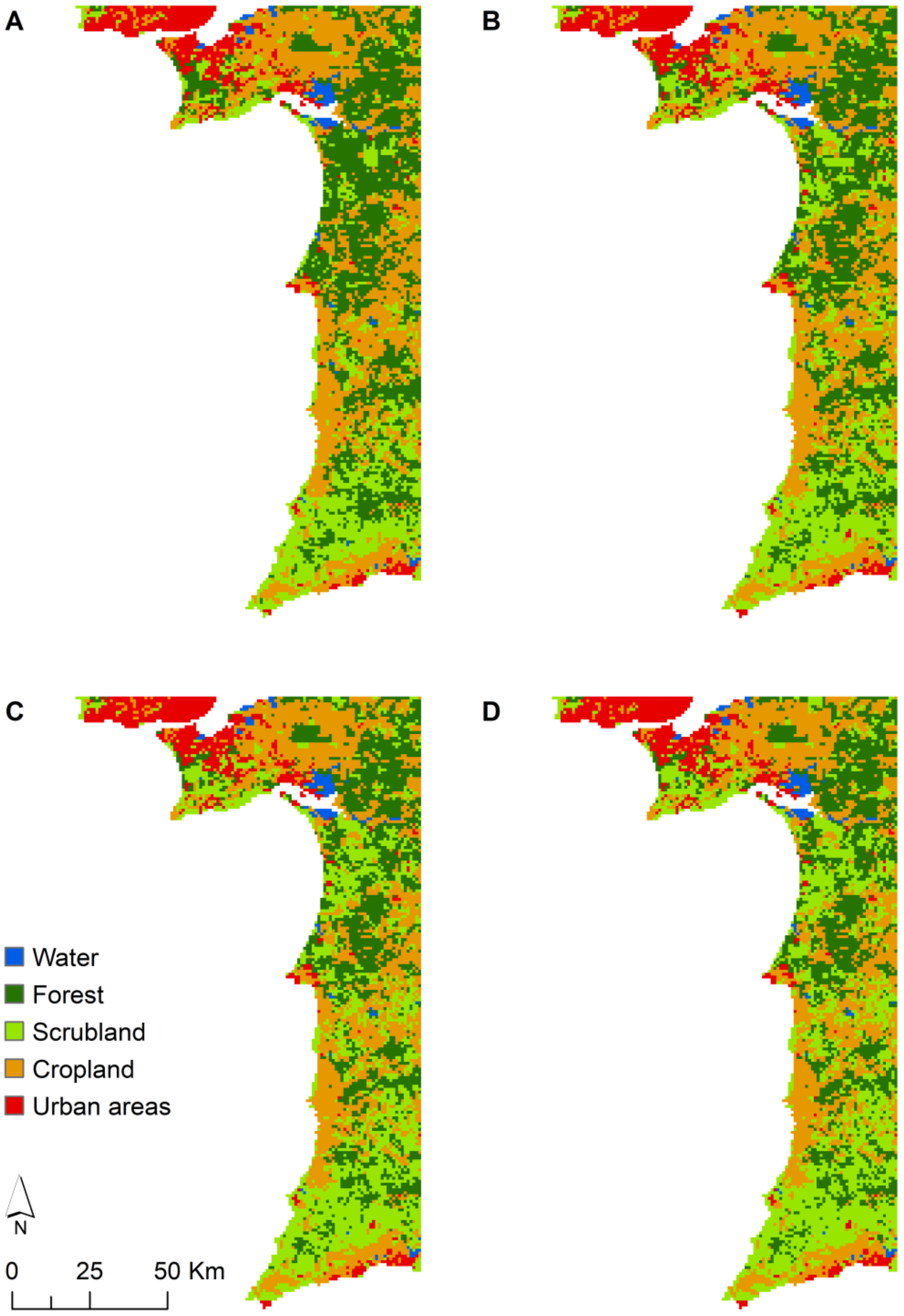

S2 Appendix-Fig. Land use maps for different periods in the study area. The CORINE Land Cover [1] maps for 2006 (A) and 2018 (B) were used to produce the simulations for 2050 (C) and 2070 (D). This figure was assembled using ArcGIS Desktop v10.8.1 [2].

S2 Appendix-Table C. Proportion of cells of each land use category for different periods in the study area, calculated from the maps depicted in S2 Appendix-Fig.

Year	2006	2018	2050	2070
Water	1.62	1.61	1.61	1.61
Forest	32.90	29.27	25.83	25.07
Scrubland	21.36	25.22	30.41	32.16
Cropland	35.67	34.90	33.03	31.97
Urban areas	8.46	9.01	9.12	9.20

References

1. European Union. Copernicus Land Monitoring Service. 2021 [cited 27 Jun 2021]. Available from: https://land.copernicus.eu/pan-european/corine-land-cover

2. ESRI. ArcGIS Desktop. Redlands, CA: Environmental Systems Research Institute; 2020.

3. Liang X, Guan Q, Clarke KC, Liu S, Wang B, Yao Y. Understanding the drivers of sustainable land expansion using a patch-generating land use simulation (PLUS) model: A case study in Wuhan, China. Computers, Environment and Urban Systems. 2021;85: 101569. doi: 10.1016/j.compenvurbsys.2020.101569

4. Hijmans RJ, Cameron SE, Parra JL, Jones PG, Jarvis A. Very high resolution interpolated climate surfaces for global land areas. International Journal of Climatology. 2005;25: 1965–1978. doi: 10.1002/joc.1276

5. Fick SE, Hijmans RJ. WorldClim 2: new 1-km spatial resolution climate surfaces for global land areas. International Journal of Climatology. 2017;37: 4302–4315. doi: 10.1002/joc.5086
