# Supplementary material for: The role of littoral cliffs in the niche delimitation on a microendemic plant facing climate change
Source: PLoS One. 2021 Oct 22;16(10):e0258976. doi: 10.1371/journal.pone.0258976 (PMC8535191; doi:10.1371/journal.pone.0258976)
Supplement: S1 Table — Total set of 102 distribution records, 38 of which selected to be used as input in the Ecological Niche Model. (PDF) [file pone.0258976.s003.pdf]

Latitude	Longitude	Used as input in the model	Source
36.996	-8.949	Yes	GBIF.org [1,2]
37.005	-8.932	No	
37.007	-8.947	No	
37.014	-8.930	No	
37.017	-8.941	No	
37.018	-8.958	No	
37.022	-8.994	No	
37.024	-8.996	Yes	
37.025	-8.994	No	
37.029	-8.978	No	
37.029	-8.982	No	
37.033	-8.985	No	
37.035	-8.990	No	
37.044	-8.889	Yes	
37.074	-8.762	Yes	
37.358	-8.841	Yes	
37.520	-8.788	No	
37.523	-8.783	No	
37.524	-8.785	Yes	
37.018	-8.956	No	
37.021	-8.991	No	
37.025	-8.989	No	
37.070	-8.800	Yes	
37.076	-8.751	No	
37.090	-8.930	Yes	
37.128	-8.916	Yes	
37.192	-8.913	No	
37.004	-8.943	No	
37.041	-8.982	No	
37.300	-8.872	No	
37.040	-8.930	Yes	GBIF.es [3]
37.597	-8.817	Yes	
37.006	-8.942	No	
37.050	-8.880	No	
37.003	-8.931	No	iNaturalist [4]
37.003	-8.946	No	
37.024	-8.994	No	
37.032	-8.983	No	
37.047	-8.978	No	
37.076	-8.751	No	
37.106	-8.938	Yes	
37.169	-8.902	No	
37.169	-8.902	Yes	
37.195	-8.916	Yes	
37.319	-8.875	No	
37.320	-8.875	Yes	
37.323	-8.868	No	
37.407	-8.811	Yes	
37.502	-8.792	Yes	
37.523	-8.783	No	
37.538	-8.788	Yes	
37.598	-8.818	No	
37.005	-8.947	No	Portuguese Society of Botany [5]
37.006	-8.946	No	
37.006	-8.946	No	
37.007	-8.947	No	
37.007	-8.928	Yes	

(continuation)

Latitude	Longitude	Used as input in the model	Source
37.016	-8.955	No	Portuguese Society of Botany [5]
37.019	-8.956	No	
37.019	-8.986	No	
37.020	-8.958	Yes	
37.021	-8.931	Yes	
37.022	-8.964	No	
37.023	-8.962	No	
37.023	-8.994	No	
37.026	-8.986	No	
37.028	-8.981	No	
37.028	-8.982	No	
37.029	-8.987	No	
37.029	-8.989	No	
37.031	-8.986	No	
37.044	-8.978	Yes	
37.046	-8.979	No	
37.047	-8.871	Yes	
37.055	-8.977	No	
37.056	-8.978	No	
37.058	-8.973	Yes	
37.144	-8.917	Yes	
37.187	-8.909	No	
37.224	-8.882	Yes	
37.243	-8.849	Yes	
37.246	-8.869	Yes	
37.248	-8.867	No	
37.291	-8.848	Yes	
37.297	-8.873	Yes	
37.302	-8.812	Yes	
37.318	-8.876	No	
37.319	-8.876	No	
37.319	-8.875	No	
37.319	-8.875	No	
37.320	-8.875	No	
37.320	-8.874	No	
37.368	-8.835	No	
37.375	-8.831	Yes	
37.439	-8.800	Yes	
37.553	-8.793	Yes	
37.558	-8.792	No	
37.581	-8.810	No	
37.583	-8.812	Yes	
37.710	-8.785	Yes	
37.030	-8.980	Yes	Quintela-Sabarís et al. [6]
37.070	-8.780	Yes	

Note: These coordinate values were referenced to the World Geodetic System 1984.

References

1. GBIF.org. GBIF occurrence download. [cited 4 Sep 2020]. Available from: https://doi.org/10.15468/dl.j8brya

2. GBIF.org. GBIF occurrence download. [cited 28 Sep 2020]. Available from: https://doi.org/10.15468/dl.zdece4

3. GBIF.es. GBIF occurrence download. [cited 1 Oct 2020]. Available from: https://registros.gbif.es/occurrences/search?q=lsid%3A6437976&fq=taxon_name%3A%22Cistus%20ladanifer%20sulcatus%22.

4. iNaturalist.org. In: iNaturalist occurrence download [Internet]. [cited 24 Sep 2020]. Available from: https://www.inaturalist.org

5. Carapeto A, Clamote F, Canha P, Caraça R, Araújo PV, Porto M. *Cistus ladanifer* L. subsp. *sulcatus* (Demoly) P.Monts. In: Flora-On: Flora de Portugal Interactiva, Sociedade Portuguesa de Botânica. [Internet]. 2020 [cited 16 Oct 2020]. Available from: https://flora-on.pt/#wCistus+ladanifer+subsp.+sulcatus

6. Quintela-Sabarís C, Vendramin GG, Castro-Fernández D, Isabel Fraga M. Chloroplast DNA phylogeography of the shrub *Cistus ladanifer* L. (Cistaceae) in the highly diverse Western Mediterranean region. Plant Biology. 2011;13: 391–400. doi: 10.1111/j.1438-8677.2010.00371.x
