# Supplementary material for: The role of littoral cliffs in the niche delimitation on a microendemic plant facing climate change
Source: PLoS One. 2021 Oct 22;16(10):e0258976. doi: 10.1371/journal.pone.0258976 (PMC8535191; doi:10.1371/journal.pone.0258976)
Supplement: S2 Table — Total set of environmental variables gathered for the present study, their units, mean ± standard deviation (minimum—maximum) values for the study area, and the source to which we resorted to obtain them. (PDF) [file pone.0258976.s004.pdf]

Type	Name	Values (Mean ± Standard Deviation (Minimum - Maximum))	Scale of original source	Source
Climatic	BIO 1 – Annual Mean Temperature (ºC * 10)	165.87 ± 4.99 (125.0 - 173.0)	30 arc-seconds (approximately 1 km)	WorldClim v1.4 [1]
	BIO 2 – Mean Diurnal Range (ºC * 10)	89.07 ± 4.89 (67.0 - 97.0)		
	BIO 3 – Isothermality (%)	42.31 ± 1.42 (40.0 - 51.0)		
	BIO 4 – Temperature Seasonality (standard deviation * 100)	4112.25 ± 285.77 (2750.0 - 4537.0)		
	BIO 5 – Maximum Temperature of Warmest Month (ºC * 10)	285.96 ± 11.31 (233.0 - 304.0)		
	BIO 6 – Minimum Temperature of Coldest Month (ºC * 10)	77.61 ± 5.38 (36.0 - 93.0)		
	BIO 7 – Temperature Annual Range (ºC * 10)	208.23 ± 12.65 (158.0 - 229.0)		
	BIO 8 – Mean Temperature of Wettest Quarter (ºC * 10)	123.99 ± 4.84 (85.0 - 139.0)		
	BIO 9 – Mean Temperature of Driest Quarter (ºC * 10)	21.69 ± 0.72 (17.8 - 22.8)		
	BIO 10 – Mean Temperature of Warmest Quarter (ºC * 10)	221.37 ± 7.27 (183.0 - 233.0)		
	BIO 11 – Mean Temperature of Coldest Quarter (ºC * 10)	116.03 ± 4.88 (76.0 - 131.0)		
	BIO 12 – Annual Precipitation (mm)	60.01 ± 6.88 (45.8 - 87.3)		
	BIO 13 – Precipitation of Wettest Month (mm)	91.23 ± 9.18 (78.0 - 131.0)		
	BIO 14 – Precipitation of Driest Month (mm)	2.28 ± 1.07 (0 - 6.0)		
	BIO 15 – Precipitation Seasonality (%)	64.73 ± 2.55 (60.0 - 72.0)		
	BIO 16 – Precipitation of Wettest Quarter (mm)	269.82 ± 26.70 (219.0 - 385.0)		
	BIO 17 – Precipitation of Driest Quarter (mm)	24.15 ± 4.52 (14.0 - 42.0)		
	BIO 18 – Precipitation of Warmest Quarter (mm)	25.64 ± 4.94 (15.0 - 44.0)		
	BIO 19 – Precipitation of Coldest Quarter (mm)	260.83 ± 25.63 (208.0 - 362.0)		
Habitat	Distance to Cliffs (m)	17358.91 ± 11491.13 (0 - 48328.82)	2 m	Calculated with Baseline Builder and Cliff Feature Delineation Tools [2] in ArcGIS Desktop v10.8.1 [3]; Digital Terrain Model: Portuguese Generate Directorate of the Territory (https://www.dgterritorio.gov.pt/)

References

1. Hijmans RJ, Cameron SE, Parra JL, Jones PG, Jarvis A. Very high resolution interpolated climate surfaces for global land areas. International Journal of Climatology. 2005;25: 1965–1978. doi: 10.1002/joc.1276

2. Seymour AC, Hapke CJ, Warrick J. Cliff Feature Delineation Tool and Baseline Builder v1.0. US Geological Survey Software Release. 2020. doi: 10.5066/P9UKW7PO

3. ESRI. ArcGIS Desktop. Redlands, CA: Environmental Systems Research Institute; 2020.
