# Supplementary material for: The role of littoral cliffs in the niche delimitation on a microendemic plant facing climate change
Source: PLoS One. 2021 Oct 22;16(10):e0258976. doi: 10.1371/journal.pone.0258976 (PMC8535191; doi:10.1371/journal.pone.0258976)
Supplement: S3 Table — The current number of suitable cells is compared with the future number of suitable cells and the resulting net gain values and percentages are presented. This comparison is made for the unrestricted scenario where C. ladanifer subsp. sulcatus can disperse everywhere, contrarily to the scenario with dispersal limitation, which considers the dispersal limitation polygon, represented in Fig 7, as the exclusive area where it may be present, both currently and in the future. RCP–Representation Concentration Pathways; GCM–General Circulation Models. (PDF) [file pone.0258976.s005.pdf]

			Without Dispersal Limitation				With Dispersal Limitation			
Year	RCP	GCM	Currently suitable cells	Future suitable cells	Net gain	Net gain percentage	Currently suitable cells	Future suitable cells	Net gain	Net gain percentage
2050	4.5	ACCESS1-0	278	126	-152	-54.3%	241	113	-128	-53.8%
		BCC-CSM1-1	278	85	-193	-68.9%	241	74	-167	-70.2%
		CCSM4	278	193	-85	-30.4%	241	154	-87	-36.6%
		MIROC-ESM	278	66	-212	-75.7%	241	55	-186	-78.2%
		MRI-CGCM3	278	21	-257	-91.8%	241	21	-220	-92.4%
	8.5	ACCESS1-0	278	119	-159	-56.8%	241	104	-137	-57.6%
		BCC-CSM1-1	278	114	-164	-58.6%	241	98	-143	-60.1%
		CCSM4	278	144	-134	-47.9%	241	125	-116	-48.7%
		MIROC-ESM	278	230	-48	-17.1%	241	155	-86	-36.1%
		MRI-CGCM3	278	0	-278	-100%	241	0	-241	-100%
2070	4.5	ACCESS1-0	278	112	-166	-59.3%	241	99	-142	-59.7%
		BCC-CSM1-1	278	490	212	75.7%	241	371	130	54.6%
		CCSM4	278	166	-112	-40.0%	241	139	-102	-42.9%
		MIROC-ESM	278	171	-107	-38.2%	241	124	-117	-49.2%
		MRI-CGCM3	278	42	-236	-84.3%	241	42	-199	-83.6%
	8.5	ACCESS1-0	278	125	-153	-54.6%	241	98	-143	-60.1%
		BCC-CSM1-1	278	93	-185	-66.1%	241	78	-163	-68.5%
		CCSM4	278	126	-152	-54.3%	241	111	-130	-54.6%
		MIROC-ESM	278	294	16	5.7%	241	203	-38	-16.0%
		MRI-CGCM3	278	27	-251	-89.6%	241	27	-214	-89.9%
